# Female BMI and Body Weight Is Not Associated with Oocyte Yield and Maturation in hCG, Agonist or Dual Trigger Cycles: A Large Observational Study including 5000 Cycles

**DOI:** 10.3390/jcm12093249

**Published:** 2023-05-01

**Authors:** Valeria Donno, Sandra García-Martínez, Nikolaos P. Polyzos

**Affiliations:** 1 Department of Reproductive Medicine, Dexeus University Hospital, 08028 Barcelona, Spain; 2 Faculty of Medicine and Health Sciences, University of Ghent, 9000 Gent, Belgium

**Keywords:** ART, trigger, oocyte maturation, BMI, weight

## Abstract

Background. Triggering final oocyte maturation is a key step of ovarian stimulation. Although previous studies demonstrated a negative association between female BMI and serum hCG levels, little evidence is available regarding the association between oocyte yield and patients’ BMI. The scope of the current study was to examine whether the efficiency of the r-hCG and triptorelin to trigger final oocyte maturation may be associated with patients’ BMI or weight. Methods. This is a retrospective observational study including 5190 ovarian stimulation cycles performed between January 2019 and September 2022 in the Reproductive Medicine Department of Dexeus University Hospital. Cycles were analyzed according to the type of trigger (triptorelin vs. r-hCG vs. dual). The primary outcome measures were oocyte maturation rate (MII/oocytes) and FOI (oocytes/AFC); secondary outcomes were oocyte and MII yield. Results. Multivariable regression analysis, adjusting for confounding factors, demonstrated that BMI was not associated with oocyte maturation rate (OR: 1.00 [95%CI: 0.99; 1.01]), FOI (Beta 0.52 [95%CI: −0.49; 1.54]), number of oocytes (Beta 0.02 [95%CI: −0.08; 0.13]) or MIIs (Beta 0.01 [95%CI: −0.08; 0.10]) retrieved. Similarly, all analyses conducted considering patients’ weight failed to reveal any association. Conclusion. Our study demonstrates that, independent of the type of trigger, patients’ BMI and weight are not associated with oocyte yield, maturation, or FOI.

## 1. Introduction

Triggering final oocyte maturation is a key step of ovarian stimulation for IVF/ICSI treatment since it represents the process by which the oocyte resumes meiosis to transition from the metaphase I to the metaphase II stage of development and attains competence for fertilization by a spermatozoon [1].

Under physiological conditions, oocyte maturation is caused by the spontaneous mid-cycle surge of luteinizing hormone (LH), but in assisted human reproduction protocols it has been replaced for decades by the administration of human chorionic gonadotropin (hCG). hCG has sufficient homology to LH and is able to activate the LH receptor [2], sharing the same α subunit and 85% of the amino-acid structure of the β subunit [3], even if it has a different pharmacokinetic profile that results in longer half-life [4].

The first hCG preparations have been developed in the late 1930s [5]. For almost 40 years, urinary hCG (uhCG) was the only hormone preparation available and different doses of hCG were described in the literature, ranging from 2000 IU to 10,000 IU. According to two early RCTs [6,7], a lower dose of urinary hCG (5000 IU) has been recommended since it does not seem to influence the probability of pregnancy compared to the conventional dose (10,000 IU) [8]. However, given that urinary preparations were associated with disadvantages like uncontrolled sources and lack of purity, uhCG has been gradually replaced over the years by recombinant hCG (rhCG), with a standard dose of 250 mcg. It corresponds to 6500 IU of uhCG [9], which has been considered at least as effective as 5000 IU or 10,000 of u-hCG [10,11].

More recently, two different types of triggering have been adopted in everyday clinical practice. First of all, the introduction of the GnRH agonist (GnRHa) trigger, which has been widely adopted in freeze-all protocols, since it is considered a breakthrough towards a safer OHSS-free clinic [12]. The second one is the dual trigger, which is a combination of GnRH agonist and hCG (both administered 36 h prior to oocyte retrieval) [13].

Nevertheless, despite the different options that have been developed over the years in order to optimize the triggering of final oocyte maturation, little evidence is available regarding the actual association between the trigger’s efficacy and patients’ BMI. Previous studies evaluated the association between BMI and ART outcomes, demonstrating the need for higher doses of drugs to stimulate ovulation [14], changes in oocyte morphology [15], reduced rates of clinical pregnancy and live births, and increased rates of miscarriage [16]. Concerning trigger’s efficacy, however, several early studies have evaluated serum hCG levels following trigger with uhCG and rhCG, demonstrating a potential association between hCG dose and patients’ BMI, without however showing any association with reproductive outcomes [10,17]. Whereas, a more recent retrospective study claimed a potential effect of patients’ BMI on the oocyte yield following hCG trigger [18].

Taking into account the accumulating evidence on the novel methods of triggering final oocyte maturation and the unclear evidence on whether patients’ body weight and BMI may be associated with oocyte yield, we set out to perform a large retrospective study in order to examine whether the efficacy of the r-hCG, triptorelin (GnRH agonist) or dual trigger (combination of rhCG and GnRH agonist) to trigger final oocyte maturation is associated with female BMI or body weight. 

## 2. Materials and Methods

Study design. This is a retrospective observational study including 5190 consecutive cycles of ovarian stimulation cycles (OS) performed between January 2019–September 2022 in the Reproductive Medicine Department of Dexeus University Hospital. We included only cycles from patients aged 18 and 40 years, who planned to undergo ovarian stimulation for IVF/ICSI using their own gamete, for oocyte donation, or medical and elective fertility preservation in a GnRH antagonist or a progestin-primed ovarian stimulation (PPOS) protocol. Patients who did not meet these criteria were excluded.

Procedures. Controlled ovarian stimulation was performed with recombinant follicle-stimulating hormone (FSH) and/or human menopausal gonadotropin (HMG) at a starting dose of 150–300 IU depending on patients’ ovarian reserve and body mass index (BMI). Control of LH surge was accomplished by a flexible GnRH antagonist or PPOS protocol. Patients’ responses to stimulation were monitored using transvaginal ultrasound starting from day 6 of ovarian stimulation, the frequency of which was determined by the clinician according to the number and diameter of growing follicles. Daily administration of a GnRH antagonist (Ganirelix 0.25 mg) was started when the leading follicle was 13–14 mm in diameter and continued until the day of the trigger of the ovulation, while in PPOS protocol Desogestrel 75 mg or micronized progesterone 200 mg was administrated orally from the beginning of ovarian stimulation until trigger day. 

As soon as at least two-three follicles had reached 18 mm in diameter, ovulation was triggered with subcutaneous administration of triptorelin at the dose of 0.2 mg or 250 mcg of r-hCG, or a combination of both in a dual trigger. Oocyte retrieval was performed 36 h after trigger injection under sedation; during the procedure, all the follicles observed were aspirated.

Outcomes. Given the retrospective nature of the study and the no-uniform baseline characteristics of the cycles, we elected primary outcomes indexes taking into account the variability of ovarian reserve. Primary outcomes measures are as follows:

- oocyte maturation rate, defined as the number of MII oocytes divided by the number of oocytes retrieved per patient; and
- FOI (Follicle to Oocyte Index) defined as the ratio between the number of oocytes collected at the ovum pick-up and the number of antral follicles at the beginning of OS [19].

Secondary outcomes include the number of oocytes retrieved and the number of MII.

Ethics approval. The study has been approved by the local ethical committee. All women were treated at the Reproductive Unite in Dexeus University Hospital, Barcelona (Spain).

Statistical analysis. Continuous variables were expressed as mean and standard deviation, whereas categorical variables were expressed as frequencies and percentages. Primary outcomes of the study were rates. Oocyte maturation rate and FOI were expressed as % [95% CI]. For bivariate analysis, the ANOVA test was used to compare patient baseline characteristics and reproductive outcomes among the three groups based on trigger dose. For the primary outcome, a generalized mixed model was used, and results were expressed as odds ratios (OR) with 95% confidence interval. The cycle was added as a random effect since each cycle has more than one oocyte. Linear regression models were used for FOI, number of oocytes retrieved, and number of MII. In this model, results were expressed as a coefficient (beta) with a 95% confidence interval. All models were adjusted by BMI, type of trigger, type of treatment, and interactions between BMI and type of trigger, and age with type of treatment. Dual trigger and oocyte donation were taken as reference groups. All tests were 2-tailed; a *p*-value of <0.05 was considered statistically significant. R software (R Core Team 2019, Vienna, Austria) and IBM© SPSS© Statistics v22 (Armonk, NY, USA) were used to perform all the analyses.

## 3. Results

Patients’ baseline characteristics: Overall, 5190 ovarian stimulation cycles (3852 patients) were analyzed: 2691 (51.8%) cycles triggered with subcutaneous administration of 0.2 mg of GnRHa (triptorelin), 1110 (21.4%) with 250 mcg of r-hCG, and 1389 (26.8%) with a dual trigger (combination of both 0.2 mg triptorelin + 250 mcg of r-hCG) (Table 1). Among them, 20.6% of cycles (1071/5190) were ovarian stimulation cycles in oocyte donors, 61.5% (3191/5190) women undergoing stimulation for IVF/ICSI, and 17.8% (926/5190) women undergoing elective or medical (non-elective) fertility preservation. Patient baseline characteristics differed among groups in terms of age (respectively 32.15 ± 5.85 in the triptorelin group vs. 36.40 ± 3.27 in r-hCG vs. 36.60 ± 3.29 in dual) and ovarian reserve (17.71 ± 8.44 vs. 9.90 ± 5.56 vs. 10.34 ± 6.07) and patients’ BMI (23.43 ± 4.63 in r-hCG group vs. 22.91 ± 4.37 in dual vs. 22.82 ± 3.78 in triptorelin group) (Table 1). 

Stimulation outcomes. Oocyte yield and MII oocyte yield were different among groups, with a higher number of oocytes (15.95 ± 8.88) and MIIs (12.31 ± 7.27) in the triptorelin group as compared with cycles triggered with rhCG (7.83 ± 5.42 and 5.86 ± 4.22 respectively) or dual trigger (8.51 ± 5.70 and 6.44 ± 4.54 respectively) (Table 1). 

**Figure 1:**
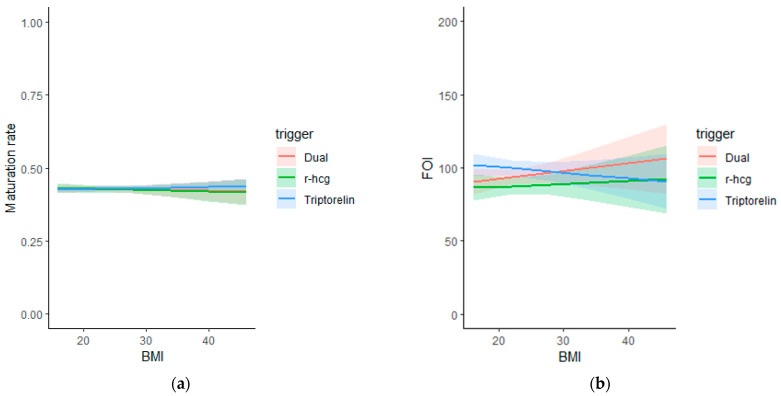
Graphical representation of the correlation between BMI and maturation rate (**a**) and FOI (**b**) in different trigger groups.

BMI and oocyte yield and maturation. A generalized mixed model, adjusting for confounding factors (age, type of trigger, and type of treatment) and adjusting for the cycle as a random effect, demonstrated that BMI was not associated with oocyte maturation rate (OR:1.00 [95% CI: 0.99;1.01]). Linear regression models adjusting for the same confounding factors, showed that BMI was not associated with FOI (Beta 0.52 [95% CI: −0.49; 1.54]) (Table 2 and Figure 1) and number of oocytes (Beta 0.02 [95% CI: −0.08; 0.13]) or with the number of MIIs (Beta 0.01 [95% CI: −0.08; 0.10]) retrieved (Table 3). Interactions between BMI and trigger groups were considered in each model.

**Figure 2:**
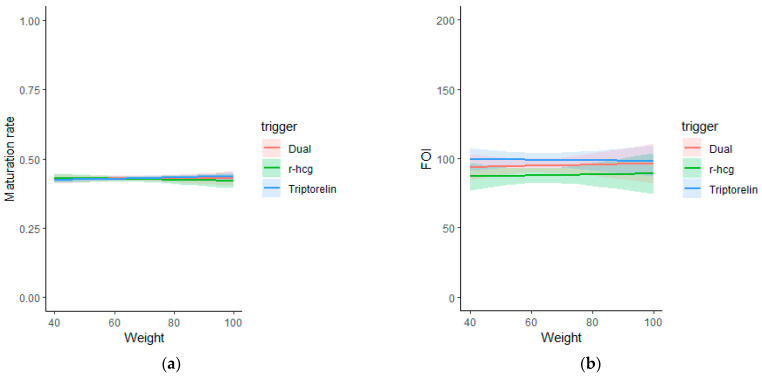
Graphical representation of the correlation between weight and maturation rate (**a**) and FOI (**b**) in different trigger groups.

Body weight and oocyte yield and maturation. Similarly, the same models were applied for body weight. These models failed to demonstrate any association between body weight with either oocyte maturation rate (OR: 1.00 [95% CI: 1.00; 1.00]), FOI (Beta 0.04 [95% CI: −0.31; 0.39]) (Table 4 and Figure 2), number of oocytes (Beta 0.01 [95% CI: −0.03; 0.04]) and number of MIIs (Beta 0.01 [95% CI: −0.02; 0.04] (Table 3). 

## 4. Discussion

To our knowledge, this is the first and the largest study evaluating the association between patients’ BMI and body weight in the efficiency of different final oocyte maturation triggering protocols. Although we see differences in oocytes and MIIs in the three groups (Table 1), these differences are justified by the clinical criteria required to enter an oocyte donation program (such as age < 35 years) and the clinical criteria for choosing the most appropriate oocyte trigger. According to that, triptorelin is normally reserved for patients who have a higher AFC and a higher number of growing follicles during ovarian stimulation in order to avoid OHSS.

Our large observational study confirms that trigger dose is not related to patients’ BMI and weight, as no significant differences have been found in maturation rate and FOI.

Over the years, the correlation between patients’ BMI or weight and trigger efficiency has been investigated focusing on hCG serum concentration after the rhCG trigger. Although a negative correlation was identified between hCG serum level and BMI after administration of 250 mcg of rhCG on trigger day, no correlation was found with reproductive outcome variables studied (number of oocytes obtained, rate of MII oocytes, fertilization rate, pregnancy rate, and live birth rate) [20,21]. These data indicate that hCG serum levels are not responsible for the failure to recover oocytes, according to the retrospective study published by Dokras et al. [22] which showed a similar number of MII oocytes in women with BMI < 25, 25–29.9 and 30–39.9 kg/m^2^.

A RCT published in 2010 comparing two doses of rhCG for final oocyte maturation (250 vs. 500 μg) in 105 overweight and obese women, failed to demonstrate any difference in terms of obtained total or MII oocytes and cycle parameters in patients with BMI > 26 kg/m^2^ [23]. Our study, in accordance with the RCT, shows that BMI does not affect oocyte and MII yield, oocyte maturation rate, and FOI in a large sample size of 5000 patients (of whom 17.3% were overweight and 6.3% obese) when the standard dose of 250 μg is used.

As far as the GnRHa trigger dose is concerned, our study is in contrast with a small retrospective study, claiming a negative association between BMI and oocyte maturity following the GnRH agonist (Triptorelin 0.2 mg) trigger [18]. In this small observational study including 113 cycles, the women with BMI < 25 Kg/m^2^ had a significantly higher number of MII oocytes as compared with women with BMI ≥ 25 Kg/m^2^. A potential explanation for the different results could be the small sample size in Lainas’ study, as well as the lack of multivariate analysis and the use of variables such as the number of oocytes and MII rather than maturation rate and FOI. On the contrary, our results are in agreement with a RCT conducted in women with low BMI in which no significant differences in the number of oocytes retrieved (18.4 ± 8.8 vs. 18.7 ± 8.9 vs. 17.8 ± 10.7) or mature oocytes (16.0 ± 8.5 vs. 15.9 ± 7.8 vs. 14.7 ± 8.4) when comparing doses ranging from 0.2 mg to 0.4 mg [24].

Finally, our study failed to find any association between BMI and maturation rate, FOI, oocyte, and MII yield following dual trigger. Although no previous study has examined the association between BMI and response in dual trigger cycles, we believe that our sample size is robust enough to exclude such an association.

In terms of clinical practice recommendation, the current large retrospective study provides robust evidence to exclude a potential association between patients’ BMI or weight and the efficiency of the rhCG and triptorelin to trigger final oocyte maturation. This suggests that clinicians should not be concerned about the triggered dose in overweight and obese women, given that even in such cases the recommended doses of 250 mcg in rhCG, of 0.2–0.4 mg in triptorelin or the combination of both in the dual trigger are sufficient to successfully induce final oocyte maturation.

The major strengths of the study are the sample size and the robust analysis performed. Although we cannot exclude the presence of residual bias despite the large series, we do believe that such a sort of bias is indeed limited owing to the adoption of multiple regression models and the indexes like maturation rate and FOI we adopted to account for confounding factors.

In conclusion, although previous small observational studies supported an association between BMI and hCG levels, our large analysis of > 5000 cycles clearly shows that the efficiency of rhCG, triptorelin, or dual trigger is not associated with the patient’s BMI and weight, as no significant differences were found in maturation rate and FOI. In this context, clinicians should not be concerned about a suboptimal response following triggering final oocyte maturation with 250 mcg of rhCG, 0.2–0.4 mg of triptorelin, or dual in overweight or obese patients.

## Figures and Tables

**Table 1:** Characteristics of the study population subdivided into three groups based on hCG ovulation trigger dose. Data are reported as mean ± SD.

Variable	Triptorelin N=2691	r-hCG N=1110	Dual N=1389	*p* Value
Woman age (y)	32.15 ± 5.85	36.40 ± 3.27	36.60 ± 3.29	<0.001
Woman height (cm)	164.20 ± 6.16	163.88 ± 6.10	164.20 ± 6.36	0.408
Woman weight (kg)	61.68 ± 10.92	63.02 ± 12.85	61.77 ± 12.49	0.016
Woman BMI (kg/m^2^)	22.82 ± 3.78	23.43 ± 4.63	22.91 ± 4.37	0.001
AFC (n)	17.71 ± 8.44	9.90 ± 5.56	10.34 ± 6.07	<0.001
Total gonadotropin dose (IU)	1923.40 ± 999.71	2427.98 ± 1077.96	2719.96 ± 1021.57	<0.001
Lenght of stimulation (d)	10.77 ± 14.57	11.46 ± 44.83	10.39 ± 10.21	0.534
Fol > 11 on trigger day (n)	17.49 ± 7.85	8.10 ± 4.74	9.10 ± 5.14	<0.001
COCs (n)	15.95 ± 8.88	7.83 ± 5.42	8.51 ± 5.70	<0.001
MII (n)	12.31 ± 7.27	5.86 ± 4.22	6.44 ± 4.54	<0.001
Maturation Rate (CI 95%)	77 (77; 78)	75 (74; 76)	76 (74; 77)	
FOI (CI 95%)	89 (87; 91)	79(76; 82)	83 (80; 86)	

**Table 2:** Association between patient’s BMI and maturation rate and FOI adjusted by type of trigger, age, and type of treatment (IVF: in vitro fertilization; FP: fertility preservation).

	Maturation Rate		FOI	
Variable	OR	[95%CI]	Beta	[95%CI]
BMI	1.00	[0.99; 1.01]	0.52	[−0.49; 1.54]
r-hCG	1.01	[0.78; 1.31]	1.24	[−32.60; 35.09]
Triptorelin	0.93	[0.76; 1.15]	25.77	[−4.40; 55.94]
Age	1.00	[1.00; 1.01]	−0.72	[−1.70; 0.26]
IVF	0.89	[0.70; 1.12]	4.21	[−36.68; 45.10]
FP	0.89	[0.59; 1.32]	41.33	[−21.25; 103.92]
BMI*hcg	1.00	[0.99; 1.01]	−0.33	[−1.76; 1.10]
BMI*triptorelin	1.00	[0.99; 1.01]	−0.90	[−2.18; 0.39]
Age*IVF	1.00	[0.99; 1.01]	0.27	[−1.03; 1.56]
Age*FP	1.00	[0.99; 1.02]	−0.86	[−2.72; 1.01]

**Table 3:** Secondary outcomes and patient BMI and weight.

	Beta	[95% CI]
BMI		
N oocytes retrieved	0.02	[−0.08; 0.13]
N MII	0.01	[−0.08; 0.10]
Weight		
N oocytes retrieved	0.01	[−0.03; 0.04]
N MII	0.01	[−0.02; 0.04]

**Table 4:** Association between patient’s weight and maturation rate and FOI adjusted by type of trigger, age, and type of treatment.

	Maturation Rate		FOI	
Variable	OR	[95%CI]	Beta	[95%CI]
Weight	1.00	[1.00; 1.00]	0.04	[−0.31; 0.39]
r-hCG	1.04	[0.82; 1.34]	−6.76	[−39.18; 25.65]
Triptorelin	0.95	[0.78; 1.15]	8.12	[−20.23; 36.46]
Age	1.00	[1.00; 1.01]	−0.79	[−1.77; 0.19]
IVF	0.90	[0.72; 1.13]	2.25	[−38.56; 43.06]
FP	0.88	[0.59; 1.31]	51.80	[−10.76; 114.36]
Weight*hcg	1.00	[1.00; 1.00]	−0.00	[−0.51; 0.38]
Weight*triptorelin	1.00	[1.00; 1.00]	−0.06	[−0.51; 0.38]
Age*IVF	1.00	[0.99; 1.01]	0.33	[−0.97; 1.62]
Age*FP	1.00	[0.99; 1.02]	−1.13	[−2.99; 0.74]

## Data Availability

Data are not available due to ethical or privacy restrictions.

## References

[1] Abbara A., Clarke S.A., Dhillo W.S. (2018). Novel Concepts for Inducing Final Oocyte Maturation in In Vitro Fertilization Treatment. Endocr. Rev..

[2] Castillo J.C., Humaidan P., Bernabéu R. (2014). Pharmaceutical Options for Triggering of Final Oocyte Maturation in ART. BioMed Res. Int..

[3] Klement A.H., Shulman A. (2017). hCG Triggering in ART: An Evolutionary Concept. Int. J. Mol. Sci..

[4] Gao F., Wang Y., Fu M., Zhang Q., Ren Y., Shen H., Han H. (2021). Effect of a “Dual Trigger” Using a GnRH Agonist and hCG on the Cumulative Live-Birth Rate for Normal Responders in GnRH-Antagonist Cycles. Front. Med..

[5] Lunenfeld B., Insler V. (1999). From Animal Gonadotrophins to Recombinant FSH.

[6] Shaltout A., Eid M., Shohayeb A. (2006). Does triggering ovulation by 5000 IU of uhCG affect ICSI outcome?. Middle East Fertil. Soc. J..

[7] Kolibianakis E.M., Papanikolaou E.G., Tournaye H., Camus M., Van Steirteghem A.C., Devroey P. (2007). Triggering final oocyte maturation using different doses of human chorionic gonadotropin: A randomized pilot study in patients with polycystic ovary syndrome treated with gonadotropin-releasing hormone antagonists and recombinant follicle-stimulating hormone. Fertil. Steril..

[8] Guideline of the European Society of Human Reproduction and Embryology. Ovarian stimulation for IVF/ICSI. October 2019. https://www.eshre.eu/Guidelines-and-Legal/Guidelines/Ovarian-Stimulation-in-IVF-ICSI.

[9] European Medicines Agency Science Medicines Health [Webpage on the Internet] Ovitrelle. https://www.ema.europa.eu/en/documents/product-information/ovitrelle-epar-product-information_en.pdf.

[10] Chang P., Kenley S., Burns T., Denton G., Currie K., DeVane G., O’dea L. (2001). Recombinant human chorionic gonadotropin (rhCG) in assisted reproductive technology: Results of a clinical trial comparing two doses of rhCG (Ovidrel) to urinary hCG (Profasi) for induction of final follicular maturation in in vitro fertilization–embryo transfer. Fertil. Steril..

[11] Ludwig M., Doody K.J., Doody K.M. (2003). Use of recombinant human chorionic gonadotropin in ovulation induction. Fertil. Steril..

[12] Devroey P., Polyzos N.P., Blockeel C. (2011). An OHSS-Free Clinic by segmentation of IVF treatment. Hum. Reprod..

[13] Haas J., Bassil R., Samara N., Zilberberg E., Mehta C., Orvieto R., Casper R.F. (2020). GnRH agonist and hCG (dual trigger) versus hCG trigger for final follicular maturation: A double-blinded, randomized controlled study. Hum. Reprod..

[14] Pandey S., Pandey S., Maheshwari A., Bhattacharya S. (2010). The impact of female obesity on the outcome of fertility treatment. J. Hum. Reprod. Sci..

[15] Depalo R., Garruti G., Totaro I., Panzarino M., Vacca M.P., Giorgino F., Selvaggi L.E. (2011). Oocyte morphological abnormalities in overweight women undergoing in vitro fertilization cycles. Gynecol. Endocrinol..

[16] Supramaniam P.R., Mittal M., McVeigh E., Lim L.N. (2018). The correlation between raised body mass index and assisted reproductive treatment outcomes: A systematic review and meta-analysis of the evidence. Reprod. Health.

[17] Chan C.C., Ng E.H., Chan M.M., Tang O.S., Lau E.Y., Yeung W.S., Ho P. (2003). Bioavailability of hCG after intramuscular or subcutaneous injection in obese and non-obese women. Hum. Reprod..

[18] Lainas G.T., Lainas T.G., Sfontouris I.A., Venetis C.A., Bosdou J.K., Chatzimeletiou A., Grimbizis G.F., Tarlatzis B.C., Kolibianakis E.M. (2020). Association between body mass index and oocyte maturation in patients triggered with GnRH agonist who are at high risk for severe ovarian hyperstimulation syndrome: An observational cohort study. Reprod. Biomed. Online.

[19] Alviggi C., Conforti A., Esteves S.C., Vallone R., Venturella R., Staiano S., Castaldo E., Andersen C.Y., De Placido G. (2018). Understanding Ovarian Hypo-Response to Exogenous Gonadotropin in Ovarian Stimulation and Its New Proposed Marker—The Follicle-To-Oocyte (FOI) Index. Front. Endocrinol..

[20] Matorras R., Meabe A., Mendoza R., Prieto B., Ramon O., Mugica J., Aspichueta F., Exposito A. (2012). Human chorionic gonadotropin (hCG) plasma levels at oocyte retrieval and IVF outcomes. J. Assist. Reprod. Genet..

[21] Mizrachi Y., Horowitz E., Farhi J., Levran D., Raziel A., Weissman A. (2018). Human chorionic gonadotropin serum levels following ovulation triggering and IVF cycle outcome. J. Assist. Reprod. Genet..

[22] Dokras A., Baredziak L., Blaine J., Syrop C., VanVoorhis B.J., Sparks A. (2006). Obstetric Outcomes after in vitro Fertilization in Obese and Morbidly Obese Women. Obstet. Gynecol..

[23] Kahraman S., Karlikaya G., Kavrut M., Karagozoglu H. (2010). A prospective, randomized, controlled study to compare two doses of recombinant human chorionic gonadotropin in serum and follicular fluid in woman with high body mass index. Fertil. Steril..

[24] Vuong T.N.L., Ho M.T., Ha T.D., Phung H.T., Huynh G.B., Humaidan P. (2016). Gonadotropin-releasing hormone agonist trigger in oocyte donors co-treated with a gonadotropin-releasing hormone antagonist: A dose-finding study. Fertil. Steril..

